# Supplementary figures and images for: Molecular patterning during the development of Phoronopsis harmeri reveals similarities to rhynchonelliform brachiopods
Source: EvoDevo. 2019 Dec 12;10:33. doi: 10.1186/s13227-019-0146-1 (PMC6907167; doi:10.1186/s13227-019-0146-1)

Brachyury

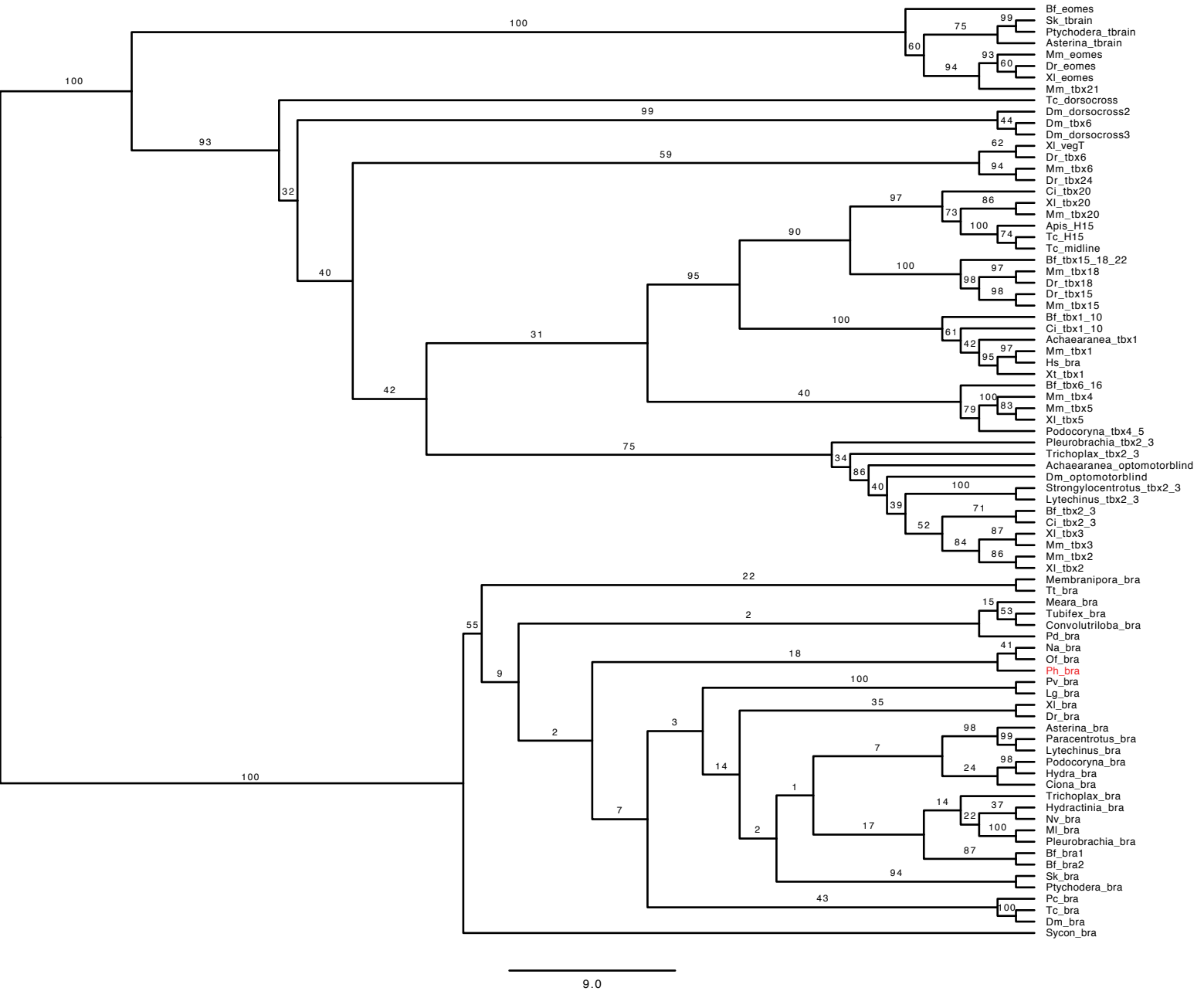

Cdx

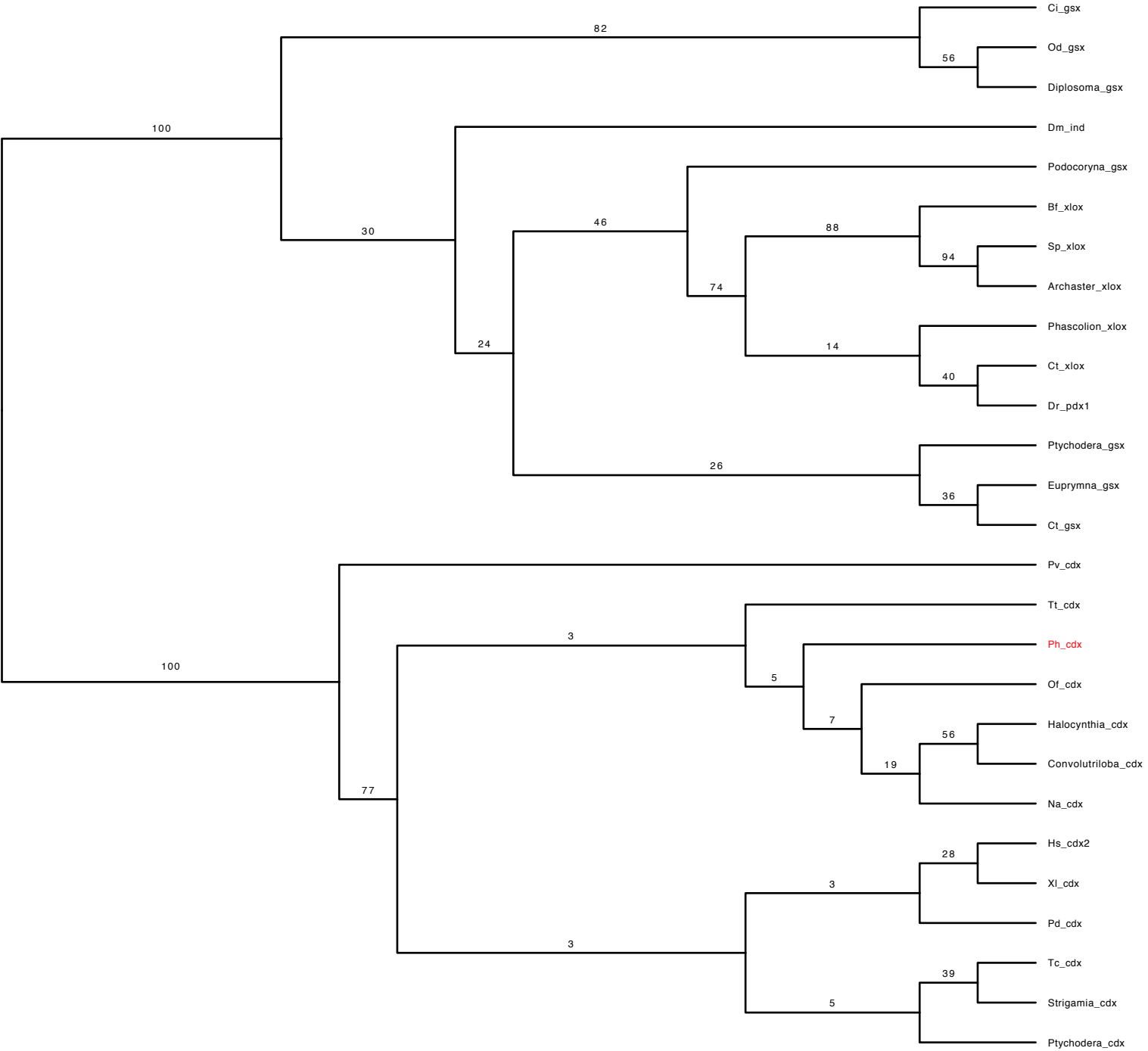

3.0

FoxA

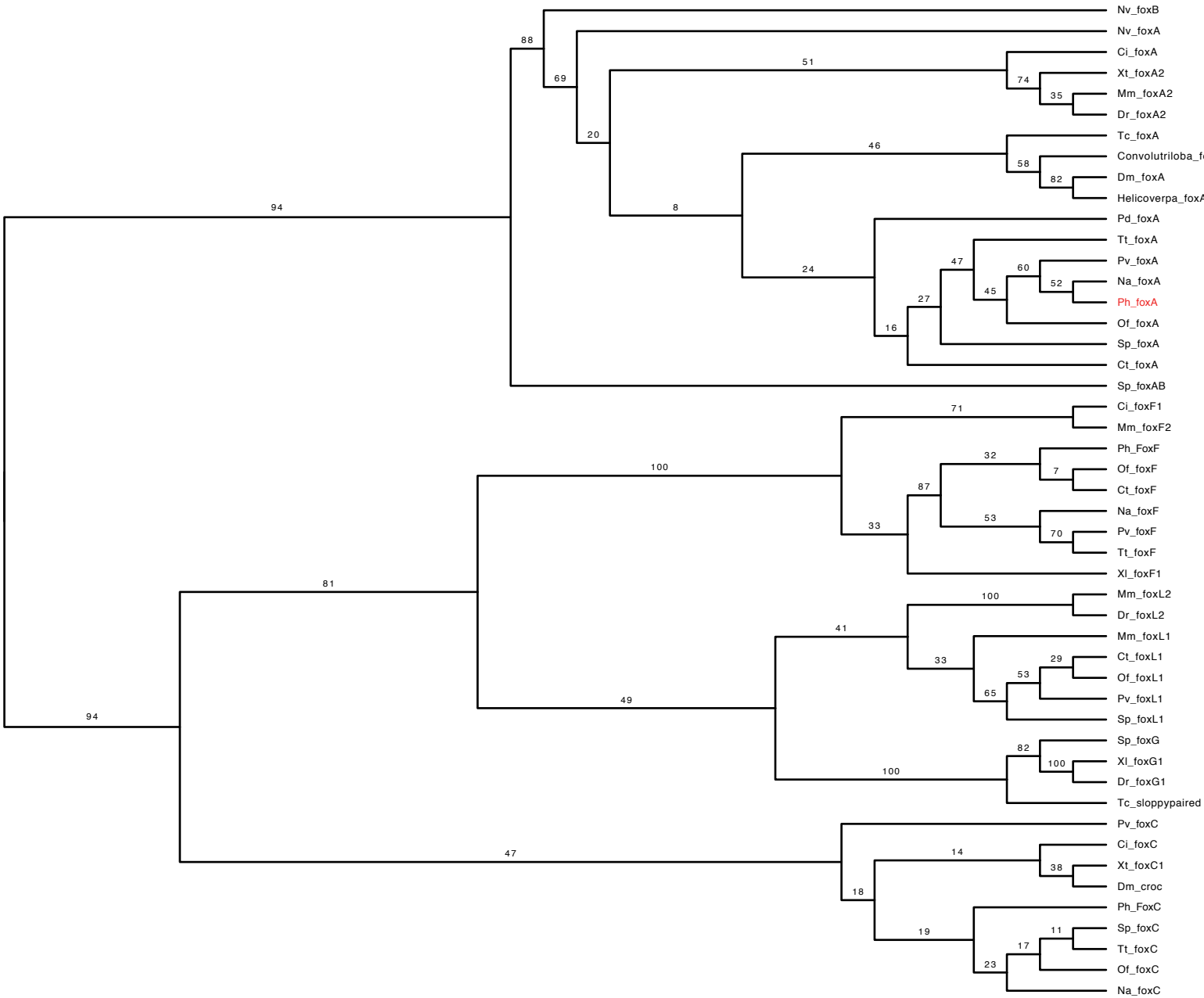

5.0

# GATA4/5/6

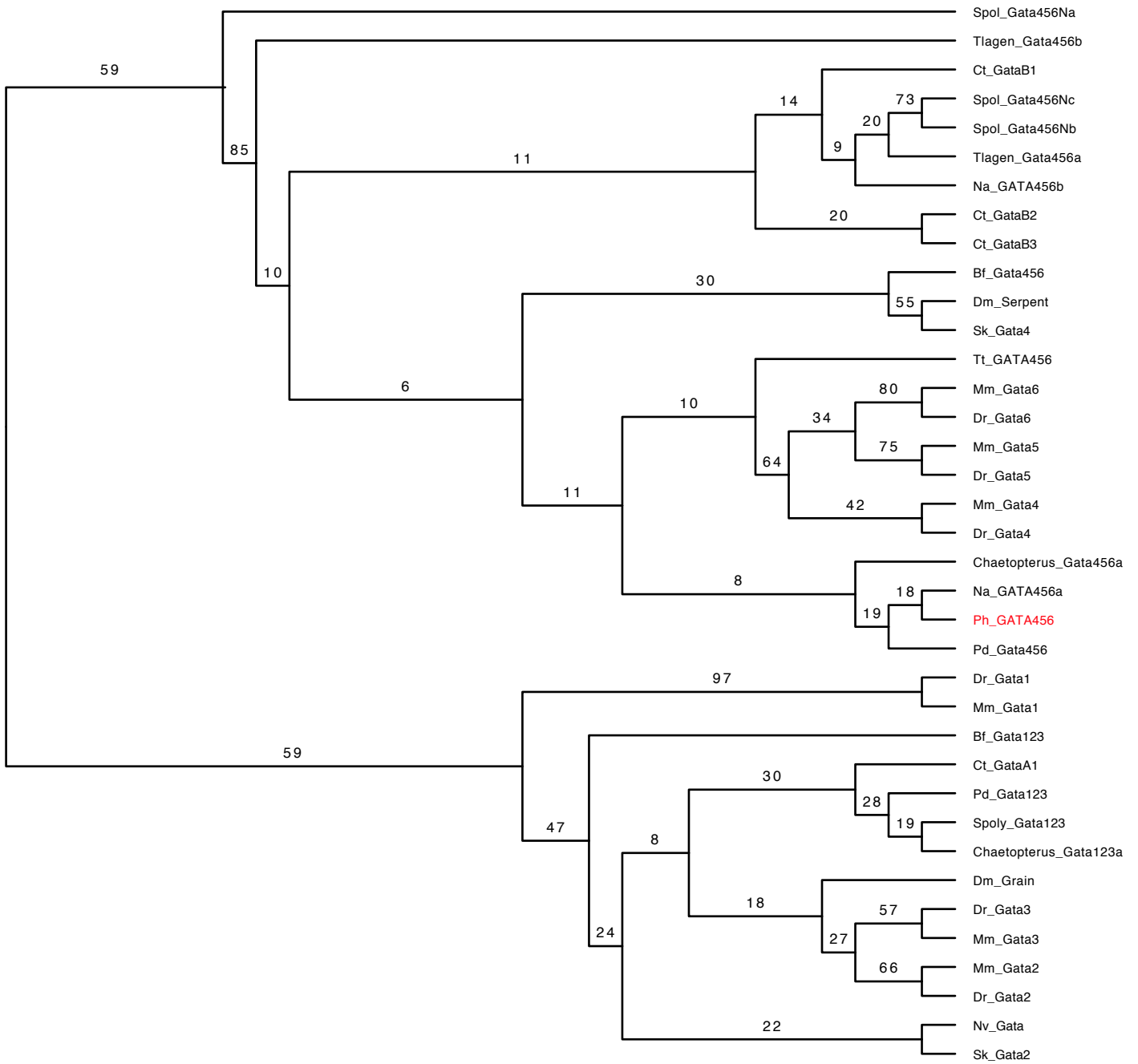

4.0

Otx/Gsc

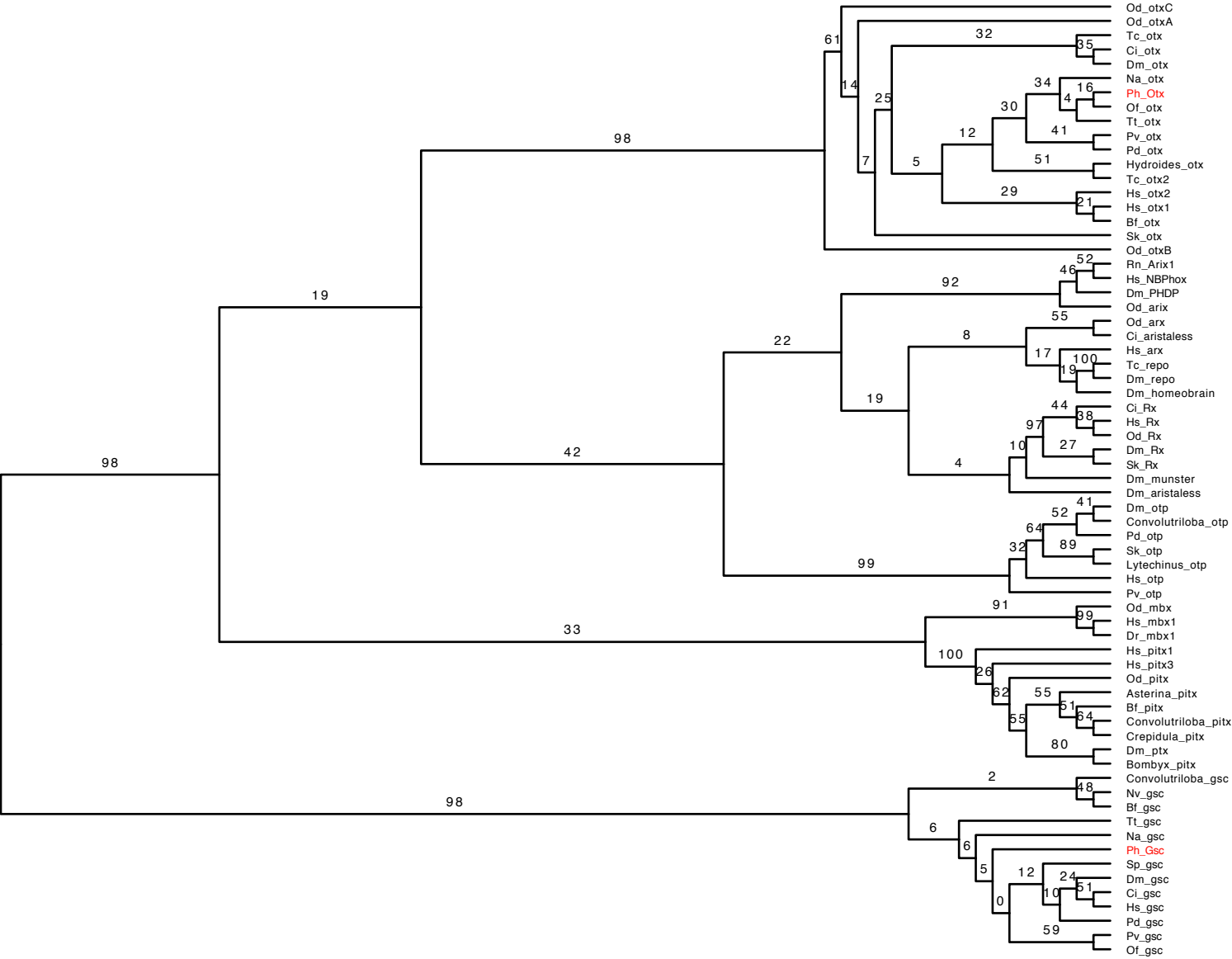

7.0

Nk2.5

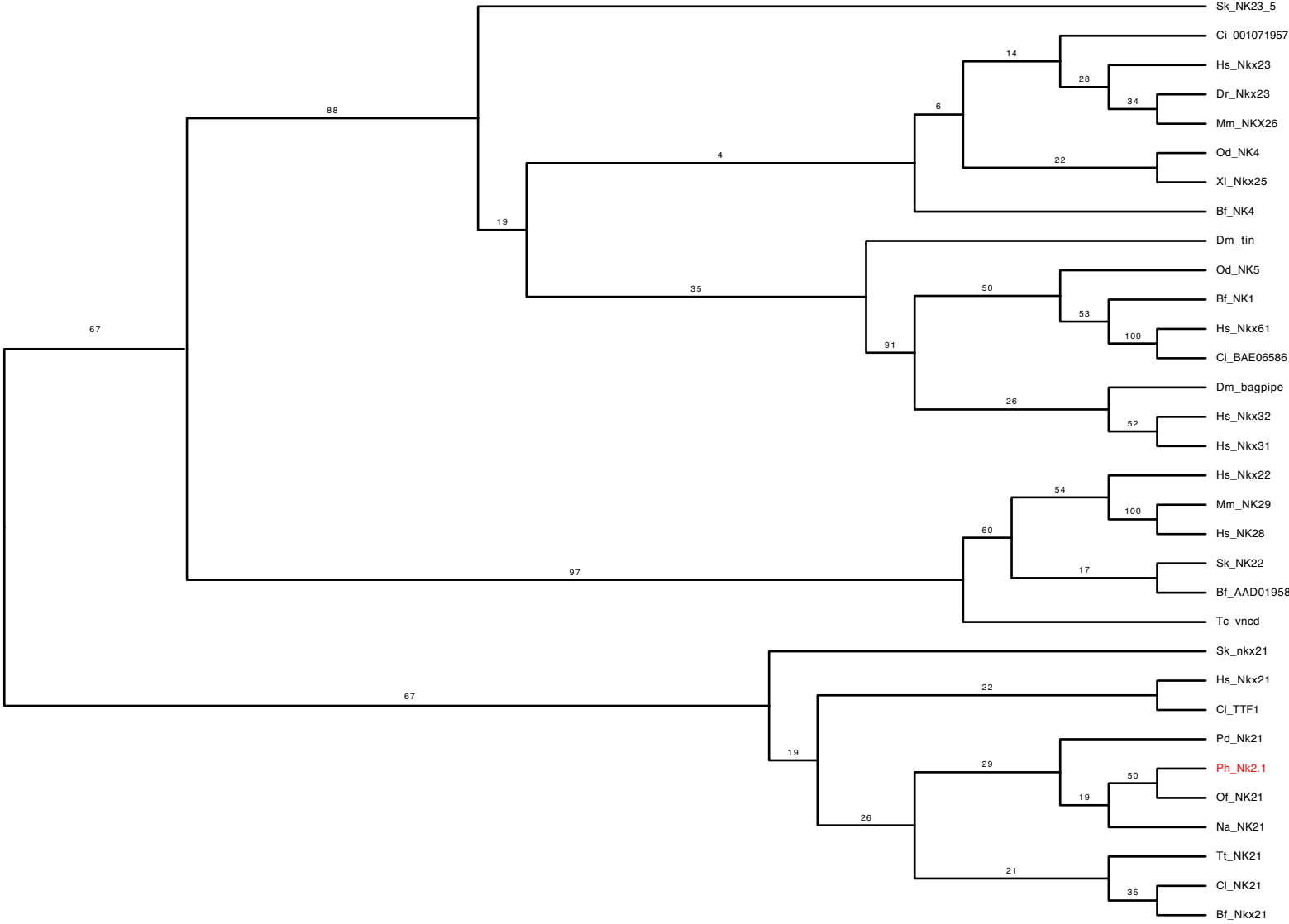

Six3/6

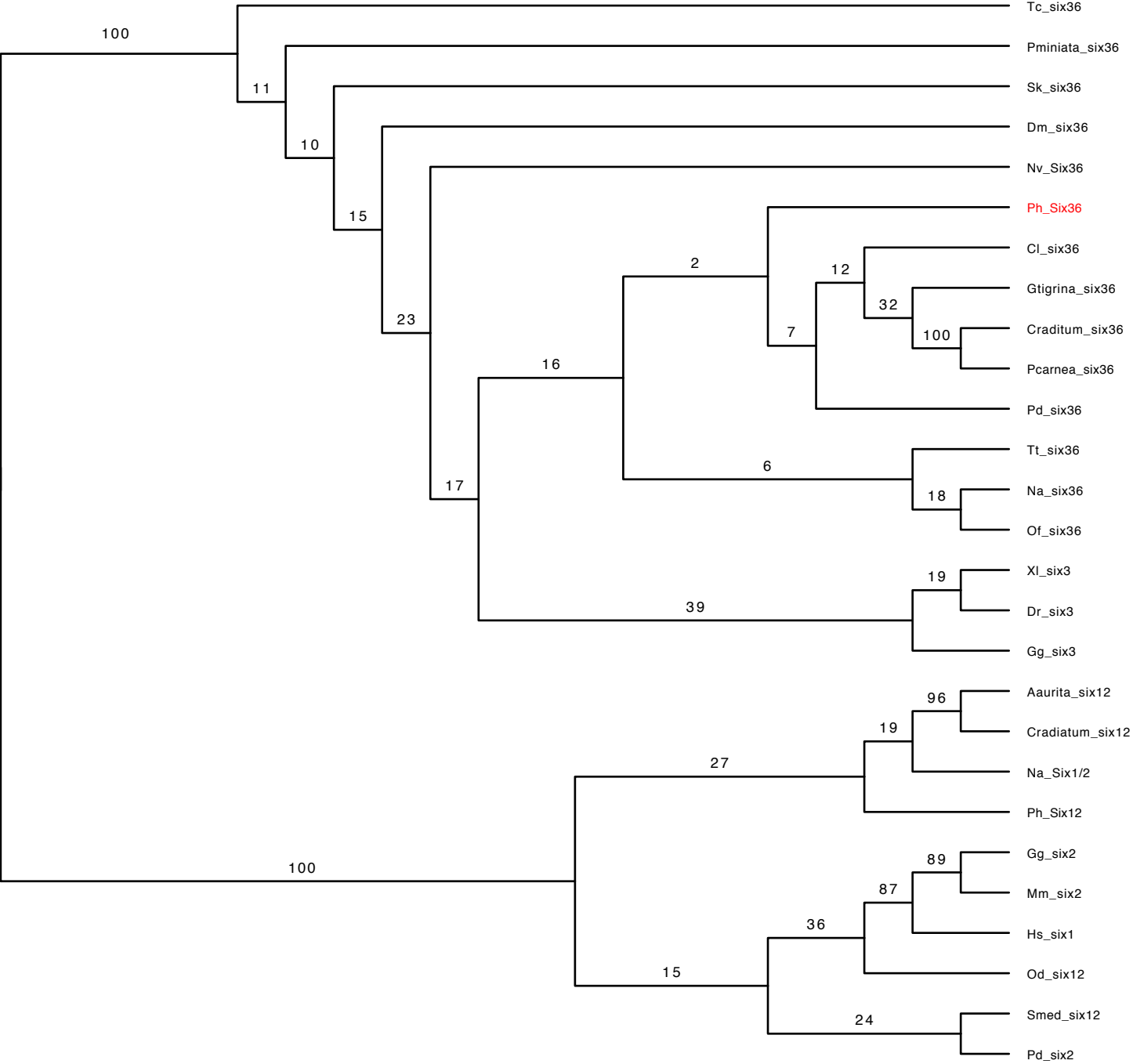

3.0

# Twist

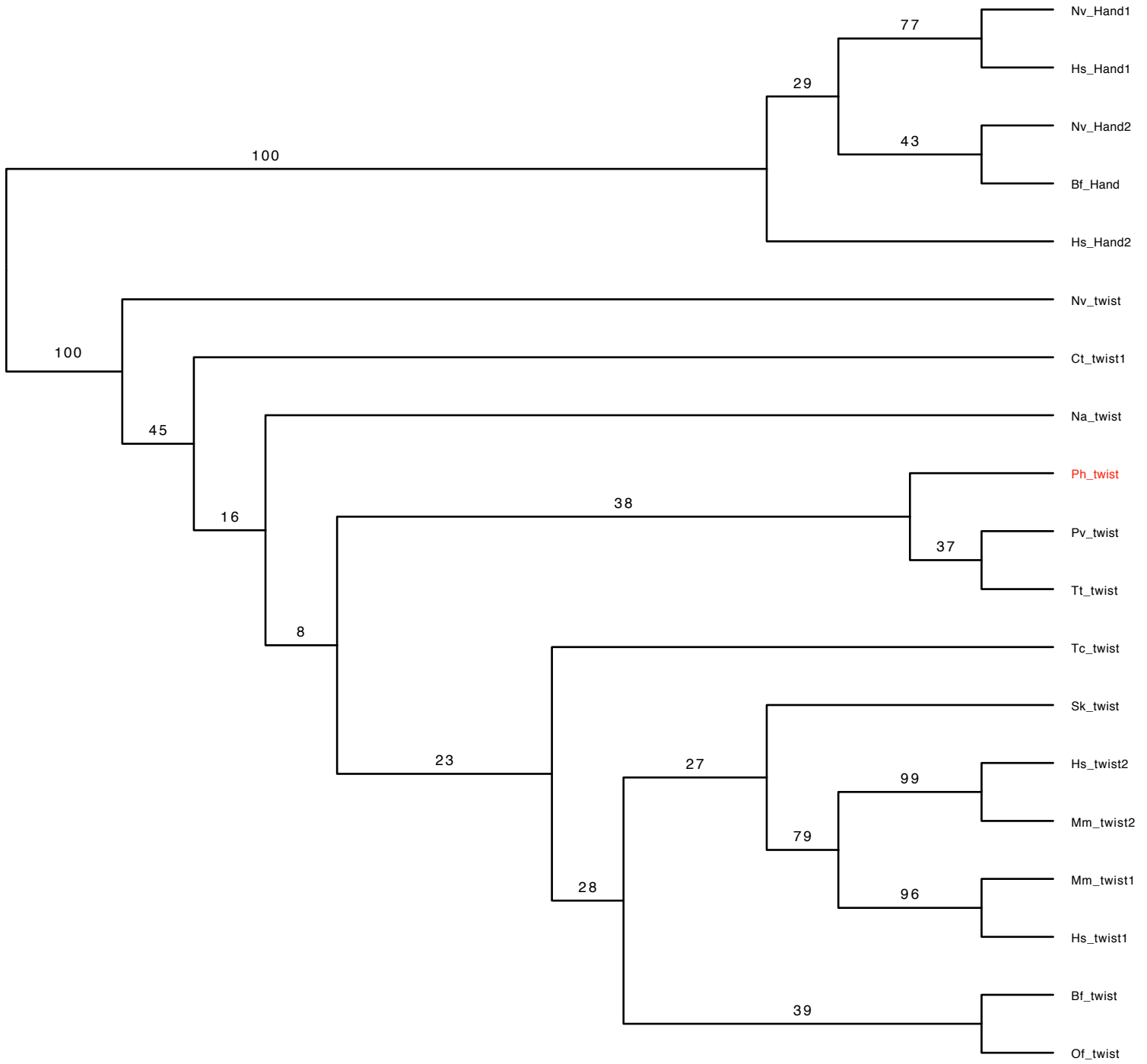

—  
0.3

Supplement: Supplementary file 1 — Additional file 1: Orthology analysis. Putative orthologous sequences of genes of interest were identified by tBLASTx search against the transcriptome of Ph. harmeri. Bayesian phylogenetic analysis is supporting orthology. Names of genes or proteins, if available, follow the name of organism(s). Ph. harmeri sequences are highlighted in red. Ph, Phoronopsis harmeri; Na, Novocrania anomala; Tt, Terebratalia transversa; Mm, Membranipora membranacea; Hs, Homo sapiens; Xl, Xenopus laevis; Xt, Xenopus tropicalis; Dr, Danio rerio; Mm, mus musculus; Gg, Gallu gallus; Sk, Saccoglossus kowalevskii; Pf, Ptychodera flava; Sp, Strongylocentrotus purpuratus; Pl, Paracentrotus lividus; Lv, Lytechinus variegatus; Am, Asterina miniata; At, Archaster typicus; Ci, Ciona intestinalis; Hl, Halocynthia roretzi; Od, Oikopleura dioica; Bf, Branchiostoma floridae; Sm, Strigamia maritima; Dm, Drosophila melanogaster; Tc, Tribolium castaneum; Lg, Lottia gigantea; Euprymna; Cf, Crepidula fornicata; Ml, Macrostomum lignano; Sm, Schmidtea mediterranea; Spoly, Schmidtea polychroa; Pv, Prostheceraeus vittatus; Gt, Girardia tigrina; Ct, Capitella teleta; Of, Owenia fusiformis; Pd, Platynereis dumerilii; He, Hydroides elegans; Tt, Tubifex tubifex; Chaetopterus; Phascolion; Ap, Apis mellifera; Pc, Priapulus caudatus; Achaearanea; Bm, Bombyx mori; Ha, Helicoverpa armigera; Nv, Nematostella vectensis; Hydractinia; Hydra; Cr, Cladonema radiatum; Aa, Aurelia aurita; Pc, Podocoryna carnea; Ms, Meara stichopi; Cm, Convolutriloba macropyga; Ta, Trichoplax adhaerens; Sc, Sycon ciliatum; Pb, Pleurobrachia bachei; Diplosoma. [file 13227_2019_146_MOESM1_ESM.pdf]
